# Prognostic Value of the Extent of Lymphadenectomy for Overall Survival Among Patients with Non-muscle Invasive Bladder Cancer, A Retrospective Cohort Study

**DOI:** 10.1245/s10434-025-17654-6

**Published:** 2025-07-10

**Authors:** Jiaxiang Ji, Chin-Hui Lai, Shicong Lai, Mingrui Wang, Haopu Hu, Runfeng Ni, Xiaolong Bian, Tao Xu, Hao Hu

**Affiliations:** 1 Department of Urology, Peking University People’s Hospital, Beijing, China; 2 The Institute of Applied Lithotripsy Technology, Peking University, Beijing, China

**Keywords:** Non-muscle invasive bladder cancer (NMIBC), Cystectomy, Extent of lymphadenectomy, Threshold, Propensity score matching (PSM)

## Abstract

**Background:**

The purpose of this study was to find out the impact of lymph node dissection (LND) on the prognosis of patients undergoing cystectomy for non-muscle invasive bladder cancer (NMIBC) and determine the minimum number of lymph nodes (LNs) for resection.

**Patients and Methods:**

Patients with NMIBC undergoing cystectomy between 2004 and 2015 were retrieved from the Surveillance, Epidemiology, and End Results (SEER) database. Propensity score matching and multivariate Cox regression analyses were performed to mitigate the influence of selection bias and control for potential confounding variables.

**Results:**

Of 1950 patients with NMIBC, 1353 underwent LND, of which 58 (4.2%) exhibited positive LNs. LND were associated with better prognosis in the whole cohort (*P* < 0.001). Patients who had positive lymph nodes were found to have the worst prognosis (*P* < 0.001). Restricted cubic spline (RCS) found that patients with at least 8 LNs examined show longer survival than those with nLNE < 8, which was further validated in multivariable Cox regression [HR 0.66 (0.59, 0.76)]. Subgroup analysis indicated that nLNE ≥ 8 was associated with significantly improved overall survival in T1 and Tis patients both before and after PSM, while for Ta patients, the impact of LND or nLNE ≥ 8 on prognosis was insignificant. Additionally, for patients with negative lymph node, nLNE ≥ 8 still resulted in better prognosis [HR 0.73 (0.61, 0.87)]. Furthermore, sensitivity analyses replicated the same results in patients more than 80 years old and found that patients with nLNE ≥ 8 demonstrated superior prognosis than those with less than 8 lymph nodes retrieved.

**Conclusions:**

Among patients with NMIBC, it is crucial to remove eight or more lymph node effectively during cystectomy, except in Ta subgroup.

**Supplementary Information:**

The online version contains supplementary material available at 10.1245/s10434-025-17654-6.

Globally, bladder cancer (BCa) ranks among the top ten most common cancers, with 550,000 new cases reported each year, 75% of which are non-muscle invasive bladder cancer (NMIBC).^1^ Transurethral resection of bladder tumor (TURBT) is the standard-of-care treatment of NMIBC. However, cystectomy remains an integral component in comprehensive treatment modalities. For patients at very high risk of progression, such as those with lymphovascular invasion (LVI) or prostatic urethral involvement of tumor, the National Comprehensive Cancer Network (NCCN) guideline recommends upfront cystectomy.^2^ In other circumstances, such as technically unresectable tumor under TURBT, urologists also tend to perform cystectomy.

While cystectomy is an important treatment modality for patients with high-risk NMIBC, the debate among urologists regarding the extent of lymphadenectomy continues. The importance of lymph node dissection (LND) is often underestimated in the treatment of NMIBC, which is usually regarded as less invasive in nature and easily curable by sole cystectomy, in contrast to its well-established role during cystectomy in the treatment of MIBC. However, according to a study of patients with NCDB, 10.6% of patients with NMIBC undergoing LND during cystectomy were found to harbor positive lymph nodes (LNs).^3^ At the same time, 16.6% of the whole cohort did not have any LNs examined at all.^3^ All these underscores the importance of LND in NMIBC patients.

LNs metastases play a significant role in the progression of bladder cancer. The American Urological Association, NCCN, and European Association of Urology (EAU) guidelines all recommend template-based lymph node dissection (LND) during radical cystectomy (RC) for muscle-invasive bladder cancer (MIBC).^4–6^ Although LN metastases were once considered rare in early stage bladder cancer cases, studies have shown that the prevalence of LN metastases in patients with NMIBC can range from 7.9% to 10.6% on the basis of various histopathological characteristics.^3,7^

During curative treatment for malignancy, the precise identification and complete removal of regional LNs is crucial for prognostic evaluation and treatment decision-making. While T staging can be precisely assessed through preoperative imaging and postoperative pathological examination, N staging depends heavily on the number of LNs retrieved during surgery. In fact, insufficient LND was found to underdiagnose advanced disease with metastatic LNs, and cause phenomenon of stage migration.^4^ Various standards of minimal number of lymph nodes examined (nLNE) during radical surgery have been proposed in other malignancies. In colon–rectal cancer, it is recommended that at least 12 negative LNs are needed to confirm the absence of nodal spread.^8^ In esophageal cancer, nodal yield < 15 is associated with reduced survival.^5^

Previous research has shown that in individuals undergoing radical cystectomy for muscle-invasive disease, a thorough and standardized lymph node dissection (LND) can significantly enhance outcomes, including reducing rates of local recurrence, distant metastases, and improving both cancer-specific and overall survival.^9^ However, the role of LND has not been fully established to date in patients with NMIBC, which is theoretically more localized and less malignant.

The aim of this study is to (1) assess the frequency of lymph nodes metastases; (2) examine the correlation between the extent of LND and patients’ prognosis; and (3) identify the minimal number of LNs that should be removed.

## Patients and Methods

### Study Population and Data Processing

This retrospective cohort is based on data from the U.S. Surveillance, Epidemiology, and End Results (SEER) Program database, which can be found at https://seer.cancer.gov/data/. The SEER database collects information on cancer incidence from population-based cancer registries, including details such as patient demographics, primary tumor site, tumor morphology, stage at diagnosis, and patient follow-up for vital status.

In our research, we enrolled patients with bladder cancer in the SEER cohort between 2004 and 2015 with “Primary Site—labeled-bladder” variable as the identifier. We further refined our selection to include only those patients who underwent cystectomy, were at T1/Ta/Tis stage, survived for at least 3 months post-operation, with known number of lymph nodes examined (nLNE), and had active follow-up post-surgery to monitor outcomes. Patients with distant metastasis, unknown cause of death, received neoadjuvant therapy, or with any other unclear clinical information were excluded from our study.

We extracted demographic, diagnostic, and survival data, such as sex, age at diagnosis, marital status, race, year of diagnosis, grade, histology, number of lymph nodes examined, number of positive lymph nodes, survival of status, and survival time.

Since SEER data are publicly accessible and deidentified, this study did not require informed consent. However, the ethical standards of institutional and national research committee oversight were maintained. The study was conducted in compliance with the 1964 Declaration of Helsinki and reported in accordance with the STROCSS criteria. It was registered in the Chinese Clinical Trial Registry (https://www.chictr.org.cn) (ChiCTR2400094409).

### Outcomes

The primary endpoint for survival analysis was overall survival (OS). OS refers to the time between initial NMIBC diagnosis and death time from any cause or last follow-up time. The secondary endpoint was cancer-specific survival (CSS). CSS refers to the time between initial diagnosis and death time from NMIBC.

### Statistical Analyses

In this research, nLNE was utilized to determine the extent of lymph node dissection. Restricted cubic spline (RCS) was applied to pinpoint the cutoff points for nLNE on the basis of OS. Survival probabilities were then computed over time for each subgroup to reassess the survival probabilities. When a noticeable disparity in survival probabilities emerged between the two new groups, the optimal cutoff point was identified.

The statistical techniques employed to determine the probability curve for survival and the correlation between survival and nLNE are laid out. The Kaplan–Meier (KM) method was employed to predict the probability curve survival. Survival probabilities between different subgroups were compared using the log-rank test. The 5- and 10-year survival probabilities, as well as the median follow-up time (which is the median observed survival time among all patients), were analyzed. Propensity score matching (PSM) was conducted according to the nearest neighbor (1:1) to reduce selection bias with matching variables including age, sex, race, marital status, histology, grade, T stage, and chemotherapy. To investigate the relationship between survival and nLNE while controlling for other secondary variables such as histology, tumor grade, histology, chemotherapy, and age at diagnosis, a multivariate Cox proportional hazard model was utilized. The magnitude of the impact of risk factors was quantified using hazard ratios (HR) along with corresponding 95% confidence intervals (CIs).

Furthermore, a sensitivity analysis was conducted to assess the reliability of the cutoff in patients exceeding 80 years old. The value is considered extreme for patients with NMIBC. All statistical computations were performed using R software (version 3.4.0).

## Results

### Demographic and Tumor Characteristics of Patients with NMIBC

A total of 1950 patients with NMIBC were included in the present study (Fig. S1A). The baseline characteristics of the study population, consisting of 1950 individuals, reveal several key demographic and clinical insights (Table 1). The majority were aged 65 years and older, constituting 61.5% of the study cohort, with the remaining 38.5% younger than 65 years. In terms of sex distribution, male participants predominated at 80.0%, while female participants comprised 20.0%. The racial composition was primarily white (88.5%), with other (6.0%) and Black (5.4%) racial groups represented to a lesser extent. Marital status at diagnosis showed that 66.9% were married. Diagnostic confirmation was predominantly through positive histology, accounting for 99.7% of cases. Regarding tumor grade, 57.0% were undifferentiated or anaplastic (grade IV), followed by poorly differentiated (grade III) at 27.8%. Histology was largely transitional cell carcinoma (TCC) at 95.0%, with variant histology (VH) at 5%. About 83.8% of tumors were classified as T1. Lymph node examination indicated that 69.4% of patients underwent LND, with 66.4% of them with negative LNs. The prevalence of LN+ in T1, Tis, and Ta was 58/1192 (4.8%), 0/31 (0%), and 0/126 (0%), respectively. In other words, all LN metastasis occurred in T1 patients. Only 16.6% of patients received adjuvant chemotherapy. These characteristics provide a comprehensive overview of the demographic and clinical profile of the study cohort.

**Table 1:** Patient demographics and baseline characteristics

Characteristic	*N* = 1950^a^
Age, years	
≥ 65	1200 (61.5%)
< 65	750 (38.5%)
Sex	
Male	1560 (80.0%)
Female	390 (20.0%)
Race	
White	1725 (88.5%)
Other	119 (6.1%)
Black	106 (5.4%)
Marital status	
Married	1305 (66.9%)
Not married	645 (33.1%)
Diagnostic confirmation	
Positive histology	1945 (99.7%)
Positive exfoliative cytology, no positive histology	5 (0.3%)
Grade	
Undifferentiated; anaplastic; grade IV	1111 (57.0%)
Poorly differentiated; grade III	543 (27.8%)
Moderately differentiated; grade II	219 (11.2%)
Well differentiated; grade I	77 (3.9%)
Histology	
TCC	1852 (95.0%)
VH	98 (5.0%)
T stage	
T1	1635 (83.8%)
Ta	244 (12.5%)
Tis	71 (3.6%)
Number of lymph node examined (nLNE)	8 (0, 20)
Lymph node dissection (LND)	
Yes	1353 (69.4%)
No	597 (30.6%)
Lymph nodes status	
Negative	1295 (66.4%)
Not examined	597 (30.6%)
Positive	58 (3.0%)
Number of positive lymph nodes (if LND conducted)	0 (0, 0)
Chemotherapy	
Yes	323 (16.6%)
No	1627 (83.4%)

^a^*n* (%); median (IQR)

Figure S1B provides the trend in the number of patients undergoing cystectomy for NMIBC in the SEER registry. Even with progress in instruments and drugs, there was still a consistent proportion of patients who received cystectomy for NMIBC.

### Impact of LND on Survival

**Fig. 1:**
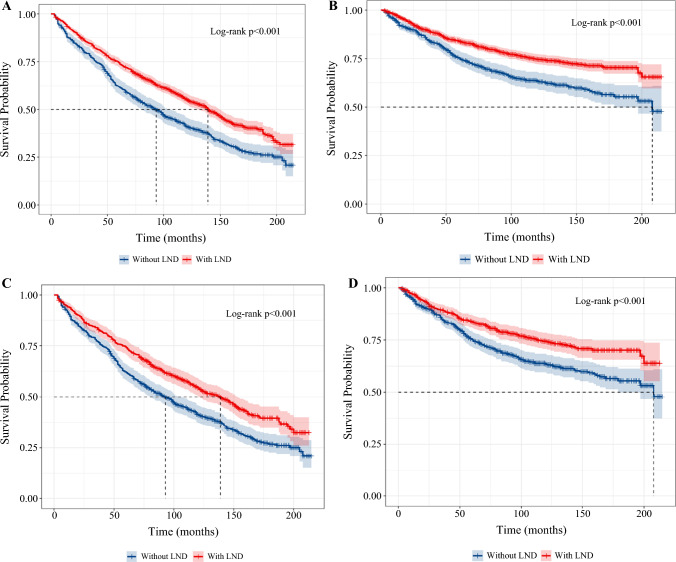
OS of patients with NMIBC with or without LND before PSM (**A**) and after PSM (**C**); CSS of patients with NMIBC with or without LND before PSM (**B**) and after PSM (**D**)

Survival was analyzed for the whole cohort with a median follow-up time of 91 months (range 3–215 months). There were 1045 deaths during the study period. The estimated 5- and 10-year OS were 74.5% and 55.3% for patients with LND and 61.8% and 41.2% for patients without LND (Fig. 1A). Furthermore, the association between LND and CSS was also investigated. The estimated 5- and 10-year CSS were 84.0% and 74.7% for patients with LND, respectively, in contrast with 75.2% and 63.4% for patients without LND, demonstrating a significant difference (*P* < 0.001) (Fig. 1B).

To evaluate the significance of LND independently, PSM analyses were performed via controlling age, sex, and other potential confounding factors (Table S1). After PSM, patients with LND were still superior in terms of OS and CSS, further validating the importance of LND in patients with NMIBC (Fig. 1C,D).

### Determination of Minimum nLNE

**Fig. 2:**
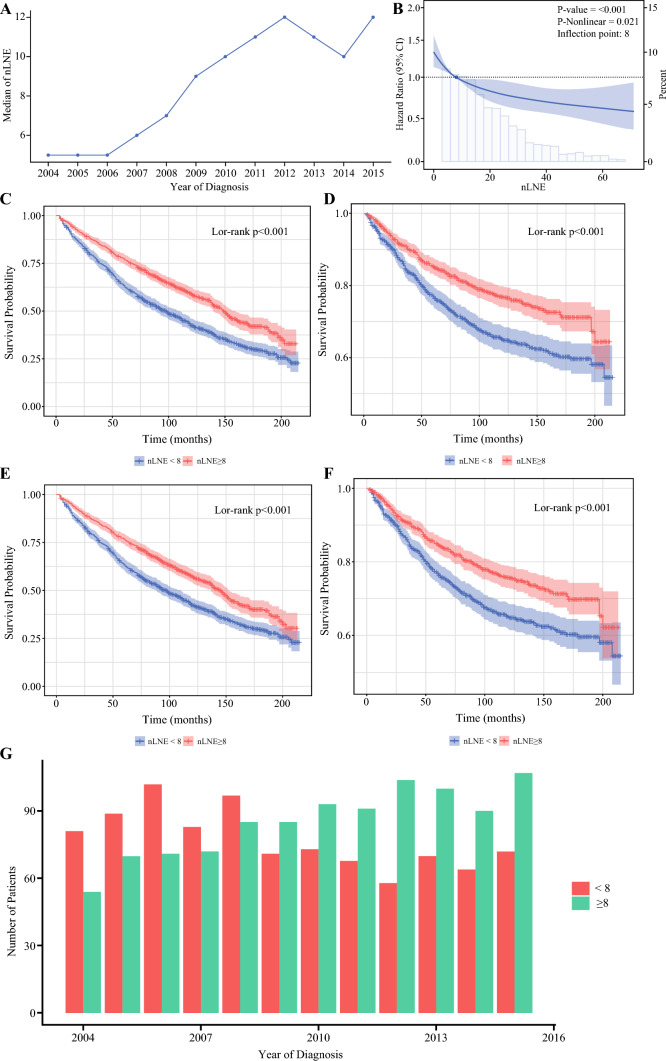
**A** Trend of median number of lymph node retrieved from 2004 to 2015; **B** relationships of HR with nLNE; nLNE approach 8 when HR is 1, KM curve of OS among patients stratified by the nLNE cutoff of 8 before PSM (**C**) and after PSM (**D**); KM curve of CSS among patients stratified by the nLNE cutoff of 8 before PSM (**E**) and after PSM (**F**); **G** trends in the number of patients undergoing adequate LND (nLNE ≥ 8) and limited LND (nLNE < 8) in the SEER registry

Figure 2A shows the trend of median nLNE from 2004 to 2015 for patients with NMIBC undergoing cystectomy, indicating an ongoing increase of LNs examined. The impact of nLNE on OS was then explored. To better clinical guidance, RCS analysis was performed on the basis of OS to find an optimal cutoff value for minimal nLNE. RCS showed that the LNs number presented a nonlinear profile (nonlinearity *P* = 0.009), and only removal of at least eight LNs can lead to significant survival benefit (Fig. 2B). The 5-year and 10-year OS rates were 77.2% and 59.0% for patients with at least eight LNs examined and 63.3% and 42.4% for those with less extensive LND (Fig. 2C). In addition, the CSS of patients with adequate LND (nLNE ≥ 8) was also superior to those with limited LND (nLNE < 8) (Fig. 2D). The benefit of adequate LND was reconfirmed after PSM with regard to OS and CSS (Table S2, Fig. 2E,F).

Figure 2G shows the trend of adequate LND and limited LND during 2004–2015, revealing a consistent improvement in LND quality.

### Univariate and Multivariate Cox Regression of Prognostic Factors for OS

**Fig. 3:**
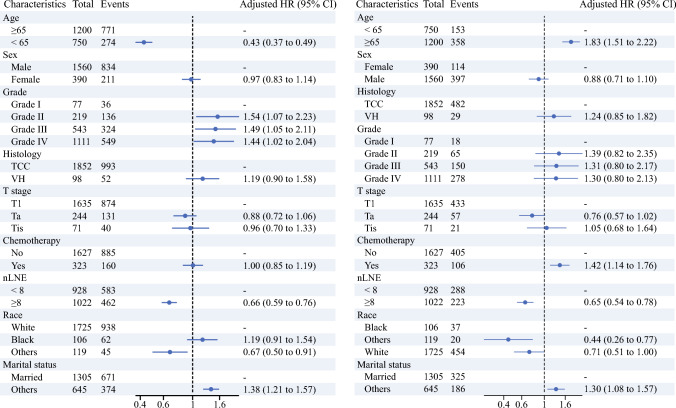
**A** Multivariable Cox regression of the whole cohort for OS; **B** multivariable Cox regression of the whole cohort for CSS

As is summarized in Fig. 3A, age (< 65 versus ≥ 65), grade (grade II/III/IV versus grade I), race (other versus white), marital status (others versus married), and nLNE (< 8 versus ≥ 8) were prognostic factors for OS in the whole cohort. Additionally, nLNE (< 8 versus ≥ 8), age (< 65 versus ≥ 65), chemotherapy (yes versus no), race (other versus Black) and marital status (other versus married) were prognostic factors for CSS (Fig. 3B).

### LN Yield and Ta/T1/Tis

The baseline characteristics of the study cohort grouped by T stage are summarized in Table 2. Significant differences were observed in histology (*P* < 0.001), where Tis had a notably lower percentage of TCC at 84.5%, compared with T1 and Ta. Grade distribution varied widely, with grade IV being most prevalent in the T1 and Tis. LND and nLNE differed significantly across various groups, with the T1 group having the highest percentage of LND completion (72.9%) and median nLNE of 9 (0, 22) (both *P* < 0.001). Lymph node status was largely negative, especially in the Ta and Tis stages, with no positive cases observed. Meanwhile, groups of Ta and Tis stages had high proportion of LND-naïve patients (48.4% and 50.7%), in contrast to T1 group (27.1%, *P* < 0.001). Chemotherapy administration was relatively consistent, administered in 12.7–16.8% of cases across T stages (*P* = 0.662). Overall, this table highlights notable differences in histology, grade, LND, and nLNE across T stages, with other characteristics being relatively consistent.

**Table 2:** Demographics and baseline characteristics of T1, Ta and Tis patients

Characteristic	T stage			*P*-value
	T1 (*n* = 1635)	Ta (*n* = 244)	Tis (*n* = 71)	
Age				0.91^b^
≥ 65	1003 (61.3%)	152 (62.3%)	45 (63.4%)	
< 65	632 (38.7%)	92 (37.7%)	26 (36.6%)	
Sex				0.21^b^
Male	1308 (80.0%)	190 (77.9%)	62 (87.3%)	
Female	327 (20.0%)	54 (22.1%)	9 (12.7%)	
Race				0.35^b^
White	1453 (88.9%)	207 (84.8%)	65 (91.5%)	
Black	87 (5.3%)	17 (7.0%)	2 (2.8%)	
Other	95 (5.8%)	20 (8.2%)	4 (5.6%)	
Marital status				0.10^b^
Married	1106 (67.6%)	149 (61.1%)	50 (70.4%)	
Other	529 (32.4%)	95 (38.9%)	21 (29.6%)	
Histology				< 0.001^c^
TCC	1552 (94.9%)	240 (98.4%)	60 (84.5%)	
VH	83 (5.1%)	4 (1.6%)	11 (15.5%)	
Grade				< 0.001^c^
Grade I	42 (2.6%)	30 (12.3%)	5 (7.0%)	
Grade II	135 (8.3%)	75 (30.7%)	9 (12.7%)	
Grade III	462 (28.3%)	65 (26.6%)	16 (22.5%)	
Grade IV	996 (60.9%)	74 (30.3%)	41 (57.7%)	
LND				< 0.001^b^
Yes	1192 (72.9%)	126 (51.6%)	35 (49.3%)	
No	443 (27.1%)	118 (48.4%)	36 (50.7%)	
nLNE	14 ± 15	8 ± 11	9 ± 14	< 0.001^d^
LN status				<0.001^d^
Positive	58 (3.5%)	0 (0.0%)	0 (0.0%)	
Negative	1134 (69.4%)	126 (51.6%)	35 (49.3%)	
NE	443 (27.1%)	118 (48.4%)	36 (50.7%)	
Chemotherapy				0.662^b^
Yes	274 (16.8%)	40 (16.4%)	9 (12.7%)	
No	1361 (83.2%)	204 (83.6%)	62 (87.3%)	

^a^*n* (%); mean ± SD

^b^Pearson’s chi-squared test

^c^Fisher’s exact test

^d^One-way ANOVA

**Fig. 4:**
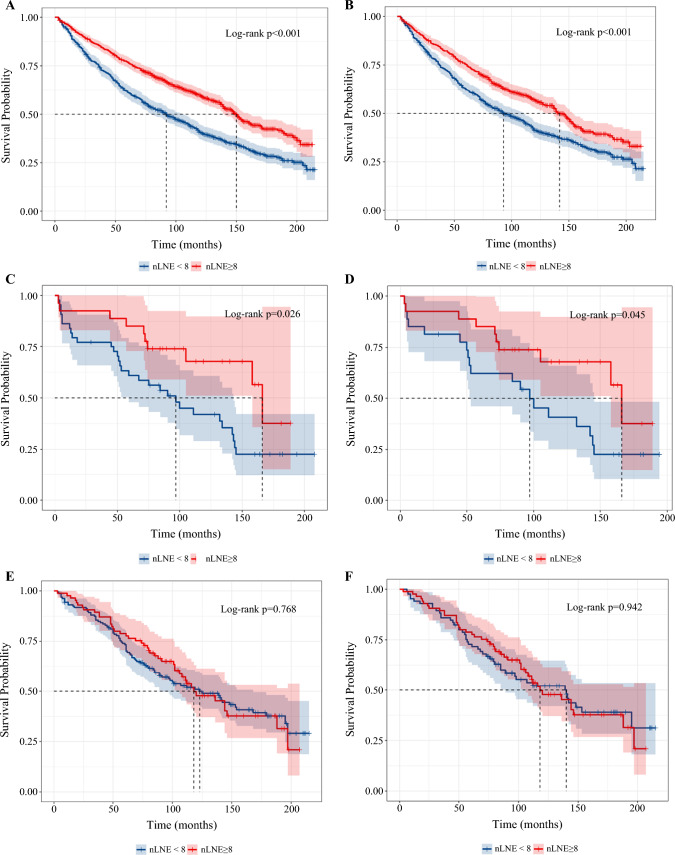
Prognostic differences between adequate and limited LND in patients with NMIBC; **A**,**B** KM curves of T1 patients before (**A**) and after PSM (**B**); **C**–**D** KM curves of Tis patients before (**C**) and after PSM (**D**); **E**,**F** KM curves of Ta patients before (**E**) and after PSM (**F**)

The KM survival curve and log-rank test showed significant differences in clinical outcomes in T1 and Tis patients with limited (< 8) and adequate (≥ 8) examined LNs before and after PSM (Table S3A,B, Fig. 4A–D). However, adequate LND failed to surpass limited LND in Ta patients (5- and 10-year OS 100% and 59.3% versus 97.8% and 64.3%) (Table S3C, Fig. 4E,F). With regard to CSS, adequate LND was also associated with improved in T1 subgroup (Fig. S2A,B). In Tis patients, the 5- and 10-year CSS were also higher in patients with at least eight LNs examined, however, without statistical significance, which might result from small cohort size (*n* = 71 before PSM and *n* = 54 after PSM) (Fig. S2C,D). CSS was comparable in Ta patients between limited and adequate LND (Fig. S2E,F).

After adjustment for well-established clinical confounders for prognosis (age, sex, grade, histology, chemotherapy, etc.), the importance of adequate LND was reconfirmed in multivariate Cox analysis for T1 (HR 0.63, *P* = 0.03) (Fig. S2G). For Tis, there still existed a trend toward survival benefit (HR 0.48, *P* = 0.05) (Fig. S2H). However, adequate LND still failed to capture a significant survival benefit in Ta patient (HR 0.71, *P* = 0.08) or even LND (HR 0.71, *P* = 0.08), which implied that LND in Ta patients did not affect overall survival (Fig. S2I).

### Extent of LND for Patients with and without Metastatic LN

**Fig. 5:**
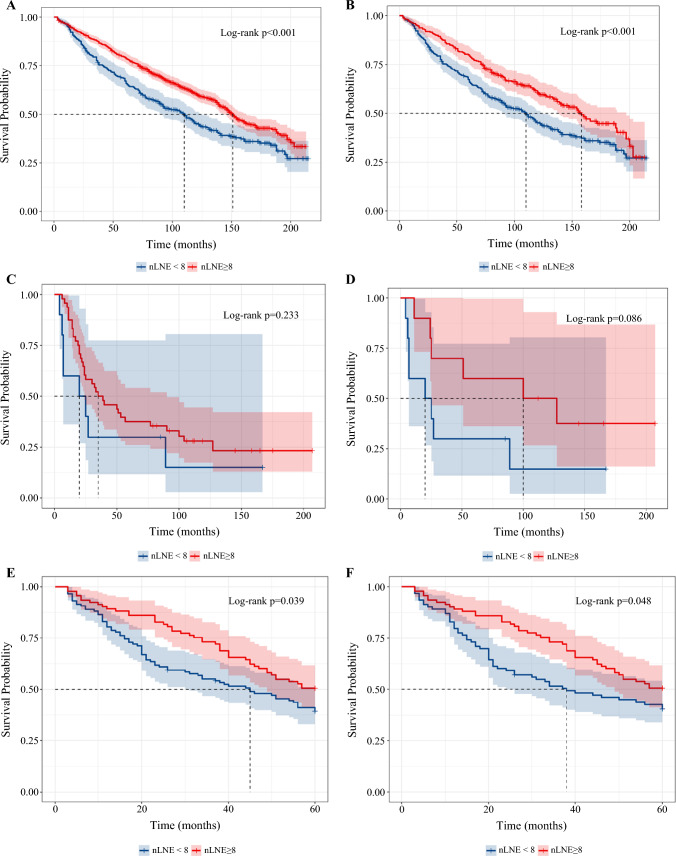
Prognostic differences between adequate and limited LND in patients with NMIBC; **A**,**B** KM curves of OS in LN− patients before (**A**) and after (**B**) PSM; **C**,**D** KM curves of OS in LN+ patients before (**C**) and after **D** PSM; **E**,**F** KM curves of OS in octogenarian patients before (**E**) and after **F** PSM

We then further our findings in patients with negative and positive LNs after LND. The KM survival curve and log-rank test showed significant differences in clinical outcomes in negative LNs subgroup between patients with limited (< 8) and adequate (≥ 8) examined LNs (5- and 10-year OS 67.1% and 45.3% versus 79.2% and 60.6%) (Fig. 5A). The benefit of adequate LND in this subgroup was also validated after PSM (Table S4A, Fig. 5B). CSS was also significantly improved by adequate LND, before and after PSM (Fig. S3A, B). Multivariable analysis proved that even without lymph node metastasis, adequate LND could still improve overall survival (HR 0.73, *P* < 0.001) (Fig. S3C).

For patients with positive LNs, there is no significant intergroup difference in overall survival based on the cutoff value of 8 before and after PSM (Table S4B, Fig. 5C, D). CSS was slightly improved by adequate LND (Fig. S3D,E). In multivariable analysis, chemotherapy and marital status were found to affect overall survival, while the extent of LND did not affect patients’ survival (Fig. S3F).

### Sensitivity Analysis

A total of 191 patients more than 80 years old were included in the sensitivity analysis, of whom 79 had at least 8 LNs removed. Although it is prudent to postulate that patients with shorter life expectation benefit less from more extensive LND, the sensitivity analysis still proved that these patients could expect improved survival with adequate LND. The 5-year overall survival and cancer-specific survival were both higher in patients with adequate LND. The benefit was consistent before and after PSM, with 5-year OS improvement of about 11.2% and 9.01%, respectively (*P* = 0.039, *P* = 0.048) (Table S4C, Fig. 5E,F). CSS was also superior in patients with adequate LND (Fig. S4A,B). However, the difference was insignificant, which may be due to the small sample size and relatively short-term follow-up.

## Discussion

LN metastasis plays a crucial role in the prognosis of patients with bladder cancer. In fact, all patients with positive LNs are classified as IV stage according to American Joint Committee on Cancer (AJCC) staging system, regardless of T stage.^10^ Lymphatic spread is complex, unpredictable, and often occurs early in the development of cancer. Due to the extensive lymphatic network in the bladder mucosa, even patients with intramucosal tumor may experience lymph node metastasis.

In the early twentieth century, Colston and Leadbetter were pioneers in challenging the prevailing belief that advanced BCa with LN metastases inevitably led to a fatal outcome and was not amenable to surgical intervention.^11^ Through an autopsy study of 98 patients in 1936, they identified 25% of patients with metastatic disease confined to pelvic LNs. This led them to propose that surgical resection could potentially offer a cure for early metastatic BCa. In 1982, Skinner was the first to demonstrate long-time survival in LN-positive patients who underwent RC along with LND.^12^ Stein recently reported 10-year recurrence-free survival of 34% in node-positive patients following RC and LND.^13^

There is currently controversy surrounding the optimal scope of LND during RC and its associated therapeutic advantages for patients with BCa. From a diagnostic standpoint, it appears sufficient to conduct a restricted LND that encompasses the obturator, external, and internal iliac regions. With the advancement of lymphadenectomy technologies and a deeper understanding of the anatomy, more extensive LND might facilitate a more precise N stage and aid clinical decisions.

The latest EAU guideline recommends a standard template-based LND in cystectomy, which includes nodal tissue cranially up to the common iliac bifurcation, with the ureter being the medial border, and including the internal iliac, obturator fossa, and external iliac nodes.^14^ Currently, there is no established threshold of nLNE in existing guidelines. Herr et al. recommended that at least 10–14 lymph nodes should be examined, which included both NMIBC and MIBC.^15^ The cutoff of ten was then applied in another study targeting NMIBC; the authors found it was only beneficial in T1 patients with more than ten lymph nodes removed, not Tis and Ta patients.^3^ In a recent study comparing limited and extended LND, the removal of at least four LNs was required in limited LND group and at least ten LNs was required in extended LND group.^16^

Recently, Gschwend et al. found that extended LND did not bring about expected survival benefit over limited LND in a MIBC-dominated cohort.^16^ Patients with extended LND did exhibit better prognosis with regard to CSS and OS, albeit without statistical significance. One potential explanation is that the sample size is rather small (*n* = 401), which is underpowered to generate a decisive conclusion. Another possible reason is that a well-designed clinical trial may not reflect real world situation. The median nLNE in the limited LDN group is 19, which far exceeds the upper threshold. By extension, the extent or even the necessity of LND in patients with less advanced disease (i.e., NMIBC) remains an open discussion. Afterall, NMIBC has much lower potential of LNs metastasis compared with MIBC.^17^ Thus, more limited LND may be sufficient for NMIBC.

Thus, our study aims to evaluate the extent of LND on the prognosis of patients with NMIBC undergoing cystectomy. Our cohort included a large number of patients with NMIBC (*N* = 1950). We found that the minimum number of LNs resected should be eight for patients with NMIBC. Such results were reconfirmed in T1 and Tis subgroups.

The clinical implications of our report are several-fold. Firstly, cystectomy remains an integral part of comprehensive treatment for NMIBC over time, without trend of decline despite advance in surgical and medical treatment. Secondly, a significant portion of patients did not receive LND or adequate LND during RC for NMIBC, although the rates of adequate LND showed improvement during the study period. Thirdly, undergoing an adequate LND was associated with improved OS even after accounting for other variables, but this benefit was primarily observed in patients with T1 and Tis disease as opposed to Ta patients. Lastly, even for patients with negative finding in LND, adequate LND is still associated with improved prognosis. These findings underscore the importance of comprehensive and meticulous LND as a crucial component of RC in patients with NMIBC.

The concept of LND originates from an approach to cancer surgery inspired by Halsted, aimed at reducing the number of cancer cells as much as possible. Despite being a fundamental aspect of radical cystectomy (RC) since its inception, it was surprising to discover that a significant number of patients underwent RC without LND. A study conducted by Abdollah et al. showed a rise in LND rates for patients with NMIBC, from 61% to 83%.^18^ Other research based on data from the National Cancer Database suggests that approximately 16.7% patients treated with RC for NMIBC did not undergo any LND at all.^3^ Throughout the period from 2004 to 2015, this study also found that only 69.4% of patients underwent RC with LND. However, the proportion of adequate LND among patients improved steadily over the time span, indicating ongoing improvement of LND quality.

Another major discovery confirmed adequate LND as an irreplaceable part for cystectomy with regard to oncological outcome. Over a relatively long median follow-up period of more than 7 years, the therapeutic and/or prognostic value of LND had a significant impact on OS. Our analysis by stage revealed that only patients with T1 and Tis disease could expect to benefit from a relatively thorough LND (≥ 8). Even after adjusting for potential confounding factors, these patients who underwent adequate LND still showed improved OS. The subgroup of Ta, by contrast, did not benefit from adequate LND or LND itself. On the one hand, it is in line with the longstanding idea that T1 and Tis are relatively more malignant and invasive than Ta. On the other hand, it reassures us that although NMIBC is long considered a rather localized disease, it is still imperative to conduct high quality LND for high-risk patients.

Consistent with this finding, the proportion of patients receiving LND in our cohort was quite different among T substages, with 72.9% (1192/1635) in T1 patients, 51.6% (126/224) in Tis patients, and 49.3% (35/71) in Ta patients. There is still much room for further improvement, especially in high-risk subgroups such as T1 and Tis.

Lastly, many people believe that LND is only meaningful in a therapeutic manner. That is to say, LND is necessary and beneficiary only when lymph node is highly likely involved. According to our study, this is not the case. Only 4.2% of patients undergoing LND were found to harbor positive LNs. However, even in patients with negative LNs, adequate LND still translated into better prognosis. The reason behind such phenomena may be that more extensive LND could more likely identify patients with true-negative LNs.^19^ According to a study on lung cancer, the accurate pN0 diagnosis depends on the number of LNs examined. The possibility of false negative findings exceeds 60% in patients with only one lymph node removed.^20^ In other words, the more LNs examined, the more likely a pathologic node-negative patient was truly free of metastatic LNs. Another reason may be that conventional hematoxylin and eosin (H&E) slides of paraffin-embedded section of LNs is insufficient for the detection of micro-metastases, which could only be identified by special immunohistochemical (IHC) staining or polymerase chain reaction (PCR).^21,22^

Micro-metastases have been shown to be present in various malignancies.^21–24^ In fact, micro-metastases do exist in bladder cancer. Kurahashi detected micro-metastases in RC specimens by real-time reverse transcriptase polymerase chain reaction (PCR) and validated its existence in 13/633 and 58/633 by different criteria.^22^ Such micro-metastases of LNs were found to prognosticate poor survival.^25,26^ Thus, removal of micro-metastatic LNs could theoretically improve prognosis, and more extensive LND, even in node-negative patients, may contribute to more complete removal of micro-metastatic LNs, and thus improve patients’ oncologic outcomes.

This study has limitations. First, some researchers believe a template-based LND is superior to the number of resected LNs.^27^ However, it is not possible to confirm its superiority in a retrospective cohort study. Second, there is no detailed and clear information regarding surgical margin, comorbidity, surgical volume, surgical approach (open versus minimally invasive), or surgical technique. Third, the indication of cystectomy is not accessible. This is quite important, as there could be some patients who are really at very high risk of recurrence and progression, such as those with persistent T1 in re-TURBT, LVI, or unresponsive to Bacillus Calmette–Guérin (BCG), and others who just personally demand a radical resection or whose tumor is technically unresectable, which are literally malignancies of different risks. However, such information also could not be retrieved in the SEER database. Last but not least, the sample size is limited, which affects the reliability of subgroup analyses. The removal of more than eight LNs failed to improve prognosis in Ta patients. However, the sample size of Ta subgroup (*n* = 244) is relatively small and may lead to insufficient statistical power. The threshold also needs further evaluation in Tis subgroup with the smallest sample size (*n* = 71).

Hence, it is important for readers to interpret the outcome of this research as a scientific advancement update, rather than a definitive guideline for surgeons, such as the suggested threshold of eight LNs. Moreover, our study is based on retrospective observational cohort, thus it is essential to validate the results through a prospective study with more sufficient information.

## Conclusions

Adequate lymphadenectomy can improve overall survival in patients with NMIBC undergoing cystectomy. This study finds that the minimal number of LNs that needs to be removed is 8, especially for T1 and Tis patients. This study adds to current knowledge regarding improving prognosis in patients with NMIBC.

## Supplementary Information

Below is the link to the electronic supplementary material.

Supplementary file1 (DOCX 1345 kb)
