# Supplementary material for: Prognostic Value of the Extent of Lymphadenectomy for Overall Survival Among Patients with Non-muscle Invasive Bladder Cancer, A Retrospective Cohort Study
Source: Ann Surg Oncol. 2025 Jul 10;32(12):9432–44. doi: 10.1245/s10434-025-17654-6 (PMC12534230; doi:10.1245/s10434-025-17654-6)
Supplement: Supplementary file 1 — Supplementary file1 (DOCX 1345 kb) [file 10434_2025_17654_MOESM1_ESM.docx]

**Table S1 Characteristics of the whole cohort before and after PSM**

| **Characteristics** | **Unmatched** | | | | | | | **Matched** | | | | | | |
| --- | --- | --- | --- | --- | --- | --- | --- | --- | --- | --- | --- | --- | --- | --- |
|  | **With LND**  (N=1,353^1^) | | | **Without LND**  (N = 597^1^) | | **p-value**^2^ | | **With LND**  **(**N= 597^1^) | | **Without LND**  (N = 597^1^) | | **p-value**^2^ | | |
| **Age** |  | | |  | | 0.241 | |  | |  | | 0.904 | | |
| ＜65 | 532 (39%) | | | 218 (37%) | |  | | 220 (37%) | | 218 (37%) | |  | | |
| ≥65 | 821 (61%) | | | 379 (63%) | |  | | 377 (63%) | | 379 (63%) | |  | | |
| **Sex** |  | | |  | | 0.041 | |  | |  | | 0.496 | | |
| Female | 254 (19%) | | | 136 (23%) | |  | | 146 (24%) | | 136 (23%) | |  | | |
| Male | 1,099 (81%) | | | 461 (77%) | |  | | 451 (76%) | | 461 (77%) | |  | | |
| **Race** |  | | |  | | 0.099 | |  | |  | | 0.669 | | |
| Black | 64 (5%) | | | 42 (7%) | |  | | 49 (8%) | | 42 (7%) | |  | | |
| Others | 86 (6%) | | | 33 (6%) | |  | | 29 (5%) | | 33 (6%) | |  | | |
| White | 1,203 (89%) | | | 522 (87%) | |  | | 519 (87%) | | 522 (87%) | |  | | |
| **Marital.status** |  | | |  | | <0.001 | |  | |  | | 0.722 | | |
| Married | 938 (69%) | | | 367 (61%) | |  | | 361 (60%) | | 367 (61%) | |  | | |
| Others | 415 (31%) | | | 230 (39%) | |  | | 236 (40%) | | 230 (39%) | |  | | |
| **Histology** |  | | |  | | 0.369 | |  | |  | | 0.800 | | |
| TCC | 1,289 (95%) | | | 563 (94%) | |  | | 565 (95%) | | 563 (94%) | |  | | |
| VH | 64 (5%) | | | 34 (6%) | |  | | 32 (5%) | | 34 (6%) | |  | | |
| **Grade** |  | | |  | | <0.001 | |  | |  | | 0.559 | | |
| Grade I | 36 (3%) | | | 41 (7%) | |  | | 34 (6%) | | 41 (7%) | |  | | |
| Grade II | 106 (8%) | | | 113 (19%) | |  | | 99 (17%) | | 113 (19%) | |  | | |
| Grade III | 354 (26%) | | | 189 (32%) | |  | | 198 (33%) | | 189 (32%) | |  | | |
| Grade IV | 857 (63%) | | | 254 (43%) | |  | | 266 (45%) | | 254 (43%) | |  | | |
| **T.stage** |  | | |  | | <0.001 | |  | |  | | 0.429 | | |
| T1 | 1,192 (88%) | | | 443 (74%) | |  | | 462 (77%) | | 443 (74%) | |  | | |
| Ta | 126 (9%) | | | 118 (20%) | |  | | 102 (17%) | | 118 (20%) | |  | | |
| Tis | 35 (3%) | | | 36 (6%) | |  | | 33 (6%) | | 36 (6%) | |  | | |
| **Chemotherapy** |  | | |  | | <0.001 | |  | |  | | 0.886 | | |
| No | 1,154 (85%) | | | 473 (79%) | |  | | 475 (80%) | | 473 (79%) | |  | | |
| Yes | 199 (15%) | | | 124 (21%) | |  | | 122 (20%) | | 124 (21%) | |  | | |
| ^1^n (%) | | | | | | | | | | | | | | |
| ^2^Pearson's Chi-squared test | | | | | | | | | | | | | | |
| **Table S2 Characteristics of the whole cohort before and after PSM stratified by nLNE of 8.** | | | | | | | | | | | | | | |
| **Characteristics** | | **Unmatched** | | | | | **Matched** | | | | | | |  |
|  |  | **nLNE≥8**,  N = 1,022^1^ | **nLNE＜8**,  N = 928^1^ | | **p-value**^2^ | | **nLNE≥8**,  N = 928^1^ | | **nLNE＜8**,  N = 928^1^ | | **p-value**^2^ | | |  |
| **Age** | |  |  | | 0.007 | |  | |  | | 0.628 | | |  |
| ＜65 | | 422 (41%) | 328 (35%) | |  | | 338 (36%) | | 328 (35%) | |  | | |  |
| ≥65 | | 600 (59%) | 600 (65%) | |  | | 590 (64%) | | 600 (65%) | |  | | |  |
| **Sex** | |  |  | | 0.081 | |  | |  | | 0.093 | | |  |
| Female | | 189 (18%) | 201 (22%) | |  | | 172 (19%) | | 201 (22%) | |  | | |  |
| Male | | 833 (82%) | 727 (78%) | |  | | 756 (81%) | | 727 (78%) | |  | | |  |
| **Race** | |  |  | | 0.084 | |  | |  | | 0.366 | | |  |
| Black | | 46 (5%) | 60 (6%) | |  | | 46 (5%) | | 60 (6%) | |  | | |  |
| Others | | 69 (7%) | 50 (5%) | |  | | 53 (6%) | | 50 (5%) | |  | | |  |
| White | | 907 (89%) | 818 (88%) | |  | | 829 (89%) | | 818 (88%) | |  | | |  |
| **Marital.status** | |  |  | | <0.001 | |  | |  | | 0.032 | | |  |
| Married | | 720 (70%) | 585 (63%) | |  | | 629 (68%) | | 585 (63%) | |  | | |  |
| Others | | 302 (30%) | 343 (37%) | |  | | 299 (32%) | | 343 (37%) | |  | | |  |
| **Histology** | |  |  | | 0.734 | |  | |  | | 0.579 | | |  |
| TCC | | 969 (95%) | 883 (95%) | |  | | 888 (96%) | | 883 (95%) | |  | | |  |
| VH | | 53 (5%) | 45 (5%) | |  | | 40 (4%) | | 45 (5%) | |  | | |  |
| **Grade** | |  |  | | <0.001 | |  | |  | | <0.001 | | |  |
| Grade I | | 26 (3%) | 51 (5%) | |  | | 26 (3%) | | 51 (5%) | |  | | |  |
| Grade II | | 75 (7%) | 144 (16%) | |  | | 75 (8%) | | 144 (16%) | |  | | |  |
| Grade III | | 239 (23%) | 304 (33%) | |  | | 239 (26%) | | 304 (33%) | |  | | |  |
| Grade IV | | 682 (67%) | 429 (46%) | |  | | 588 (63%) | | 429 (46%) | |  | | |  |
| **T.stage** | |  |  | | <0.001 | |  | |  | | <0.001 | | |  |
| T1 | | 910 (89%) | 725 (78%) | |  | | 816 (88%) | | 725 (78%) | |  | | |  |
| Ta | | 85 (8%) | 159 (17%) | |  | | 85 (9%) | | 159 (17%) | |  | | |  |
| Tis | | 27 (3%) | 44 (5%) | |  | | 27 (3%) | | 44 (5%) | |  | | |  |
| **Chemotherapy** | |  |  | | 0.443 | |  | |  | | 0.902 | | |  |
| No | | 859 (84%) | 768 (83%) | |  | | 770 (83%) | | 768 (83%) | |  | | |  |
| Yes | | 163 (16%) | 160 (17%) | |  | | 158 (17%) | | 160 (17%) | |  | | |  |
| ^1^n (%) | | | | | | | | | | | | | |  |
| ^2^Pearson's Chi-squared test | | | | | | | | | | | | | |  |
| ^1^n (%) | | | | | | | | | | | | |  |  |
| ^2^Pearson's Chi-squared test | | | | | | | | | | | | |  |  |

**Table S3A Characteristics of the T1 subgroup before and after PSM stratified by nLNE of 8.**

| **Characteristics** | **Unmatched** | | | **Matched** | | |
| --- | --- | --- | --- | --- | --- | --- |
|  | **≥8**, N = 910^1^ | **＜8**, N = 725^1^ | **p-value**^2^ | **≥8**, N = 725^1^ | **＜8**, N = 725^1^ | **p-value**^2^ |
| **Age** |  |  | 0.002 |  |  | 0.660 |
| ≥65 | 528 (58%) | 475 (66%) |  | 467 (64%) | 475 (66%) |  |
| ＜65 | 382 (42%) | 250 (34%) |  | 258 (36%) | 250 (34%) |  |
| **Sex** |  |  | 0.135 |  |  | 0.400 |
| Male | 740 (81%) | 568 (78%) |  | 581 (80%) | 568 (78%) |  |
| Female | 170 (19%) | 157 (22%) |  | 144 (20%) | 157 (22%) |  |
| **Race** |  |  | 0.108 |  |  | 0.681 |
| White | 812 (89%) | 641 (88%) |  | 651 (90%) | 641 (88%) |  |
| Others | 58 (6%) | 37 (5%) |  | 34 (5%) | 37 (5%) |  |
| Black | 40 (4%) | 47 (6%) |  | 40 (6%) | 47 (6%) |  |
| **Marital.status** |  |  | 0.009 |  |  | 0.377 |
| Married | 640 (70%) | 466 (64%) |  | 482 (66%) | 466 (64%) |  |
| Others | 270 (30%) | 259 (36%) |  | 243 (34%) | 259 (36%) |  |
| **Histology** |  |  | 0.388 |  |  | 0.804 |
| TCC | 860 (95%) | 692 (95%) |  | 690 (95%) | 692 (95%) |  |
| VH | 50 (5%) | 33 (5%) |  | 35 (5%) | 33 (5%) |  |
| **Grade** |  |  | <0.001 |  |  | <0.001 |
| Grade III | 213 (23%) | 249 (34%) |  | 211 (29%) | 249 (34%) |  |
| Grade IV | 626 (69%) | 370 (51%) |  | 443 (61%) | 370 (51%) |  |
| Grade II | 52 (6%) | 83 (11%) |  | 52 (7%) | 83 (11%) |  |
| Grade I | 19 (2%) | 23 (3%) |  | 19 (3%) | 23 (3%) |  |
| **Chemotherapy** |  |  | 0.162 |  |  | 0.535 |
| No | 768 (84%) | 593 (82%) |  | 602 (83%) | 593 (82%) |  |
| Yes | 142 (16%) | 132 (18%) |  | 123 (17%) | 132 (18%) |  |
| ^1^n (%) | | | | | | |
| ^2^Pearson's Chi-squared test | | | | | | |

**Table S3B Characteristics of the Tis subgroup before and after PSM stratified by nLNE of 8.**

| **Characteristics** | **Unmatched** | | | **Matched** | | |
| --- | --- | --- | --- | --- | --- | --- |
|  | **＜8**, N = 44^1^ | **≥8**, N = 27^1^ | **p-value**^2^ | **＜8**, N = 27^1^ | **≥8**, N = 27^1^ | **p-value**^2^ |
| **Age** |  |  | 0.954 |  |  | 0.564 |
| ≥65 | 28 (64%) | 17 (63%) |  | 19 (70%) | 17 (63%) |  |
| ＜65 | 16 (36%) | 10 (37%) |  | 8 (30%) | 10 (37%) |  |
| **Sex** |  |  | 0.286 |  |  | 0.420 |
| Female | 4 (9%) | 5 (19%) |  | 2 (7%) | 5 (19%) |  |
| Male | 40 (91%) | 22 (81%) |  | 25 (93%) | 22 (81%) |  |
| **Race** |  |  | 0.823 |  |  | >0.999 |
| White | 41 (93%) | 24 (89%) |  | 25 (93%) | 24 (89%) |  |
| Others | 2 (5%) | 2 (7%) |  | 1 (4%) | 2 (7%) |  |
| Black | 1 (2%) | 1 (4%) |  | 1 (4%) | 1 (4%) |  |
| **Marital.status** |  |  | 0.287 |  |  | 0.484 |
| Married | 29 (66%) | 21 (78%) |  | 23 (85%) | 21 (78%) |  |
| Others | 15 (34%) | 6 (22%) |  | 4 (15%) | 6 (22%) |  |
| **Histology** |  |  | 0.515 |  |  | 0.236 |
| TCC | 36 (82%) | 24 (89%) |  | 27 (100%) | 24 (89%) |  |
| VH | 8 (18%) | 3 (11%) |  | 0 (0%) | 3 (11%) |  |
| **Grade** |  |  | 0.599 |  |  | >0.999 |
| Grade III | 12 (27%) | 4 (15%) |  | 5 (19%) | 4 (15%) |  |
| Grade II | 6 (14%) | 3 (11%) |  | 3 (11%) | 3 (11%) |  |
| Grade I | 3 (7%) | 2 (7%) |  | 1 (4%) | 2 (7%) |  |
| Grade IV | 23 (52%) | 18 (67%) |  | 18 (67%) | 18 (67%) |  |
| **Chemotherapy** |  |  | 0.286 |  |  | >0.999 |
| No | 40 (91%) | 22 (81%) |  | 23 (85%) | 22 (81%) |  |
| Yes | 4 (9%) | 5 (19%) |  | 4 (15%) | 5 (19%) |  |
| ^1^n (%) | | | | | | |
| ^2^Pearson's Chi-squared test; Fisher's exact test | | | | | | |

**Table S3C Characteristics of the Tis subgroup before and after PSM stratified by nLNE of 8.**

| **Characteristics** | **Unmatched** | | | **Matched** | | |
| --- | --- | --- | --- | --- | --- | --- |
|  | **＜8**, N = 159^1^ | **≥8**, N = 85^1^ | **p-value**^2^ | **＜8**, N = 85^1^ | **≥8**, N = 85^1^ | **p-value**^2^ |
| **Age** |  |  | 0.570 |  |  | 0.873 |
| ≥65 | 97 (61%) | 55 (65%) |  | 54 (64%) | 55 (65%) |  |
| ＜65 | 62 (39%) | 30 (35%) |  | 31 (36%) | 30 (35%) |  |
| **Sex** |  |  | 0.119 |  |  | 0.551 |
| Male | 119 (75%) | 71 (84%) |  | 68 (80%) | 71 (84%) |  |
| Female | 40 (25%) | 14 (16%) |  | 17 (20%) | 14 (16%) |  |
| **Race** |  |  | 0.561 |  |  | 0.901 |
| White | 136 (86%) | 71 (84%) |  | 73 (86%) | 71 (84%) |  |
| Others | 11 (7%) | 9 (11%) |  | 8 (9%) | 9 (11%) |  |
| Black | 12 (8%) | 5 (6%) |  | 4 (5%) | 5 (6%) |  |
| **Marital.status** |  |  | 0.051 |  |  | 0.332 |
| Married | 90 (57%) | 59 (69%) |  | 53 (62%) | 59 (69%) |  |
| Others | 69 (43%) | 26 (31%) |  | 32 (38%) | 26 (31%) |  |
| **Histology** |  |  | 0.301 |  |  | >0.999 |
| TCC | 155 (97%) | 85 (100%) |  | 85 (100%) | 85 (100%) |  |
| VH | 4 (3%) | 0 (0%) |  | 0 (0%) | 0 (0%) |  |
| **Grade** |  |  | 0.001 |  |  | 0.969 |
| Grade III | 43 (27%) | 22 (26%) |  | 23 (27%) | 22 (26%) |  |
| Grade IV | 36 (23%) | 38 (45%) |  | 36 (42%) | 38 (45%) |  |
| Grade II | 55 (35%) | 20 (24%) |  | 22 (26%) | 20 (24%) |  |
| Grade I | 25 (16%) | 5 (6%) |  | 4 (5%) | 5 (6%) |  |
| **Chemotherapy** |  |  | 0.453 |  |  | 0.541 |
| No | 135 (85%) | 69 (81%) |  | 72 (85%) | 69 (81%) |  |
| Yes | 24 (15%) | 16 (19%) |  | 13 (15%) | 16 (19%) |  |
| ^1^n (%) | | | | | | |
| ^2^Pearson's Chi-squared test; Fisher's exact test | | | | | | |

**Table S4A Characteristics of the N- subgroup before and after PSM stratified by nLNE of 8.**

| **Characteristics** | **Unmatched** | | | **Matched** | | |
| --- | --- | --- | --- | --- | --- | --- |
|  | **≥8**, N = 974^1^ | **＜8**, N = 321^1^ | **p-value**^2^ | **≥8**, N = 321^1^ | **＜8**, N = 321^1^ | **p-value**^2^ |
| **Age** |  |  | 0.014 |  |  | >0.999 |
| ≥65 | 577 (59%) | 215 (67%) |  | 215 (67%) | 215 (67%) |  |
| ＜65 | 397 (41%) | 106 (33%) |  | 106 (33%) | 106 (33%) |  |
| **Sex** |  |  | 0.739 |  |  | 0.920 |
| Male | 797 (82%) | 260 (81%) |  | 259 (81%) | 260 (81%) |  |
| Female | 177 (18%) | 61 (19%) |  | 62 (19%) | 61 (19%) |  |
| **Race** |  |  | 0.428 |  |  | 0.966 |
| White | 863 (89%) | 288 (90%) |  | 290 (90%) | 288 (90%) |  |
| Others | 67 (7%) | 16 (5%) |  | 15 (5%) | 16 (5%) |  |
| Black | 44 (5%) | 17 (5%) |  | 16 (5%) | 17 (5%) |  |
| **Marital.status** |  |  | 0.090 |  |  | 0.509 |
| Married | 692 (71%) | 212 (66%) |  | 204 (64%) | 212 (66%) |  |
| Others | 282 (29%) | 109 (34%) |  | 117 (36%) | 109 (34%) |  |
| **Histology** |  |  | 0.188 |  |  | 0.824 |
| TCC | 923 (95%) | 310 (97%) |  | 311 (97%) | 310 (97%) |  |
| VH | 51 (5%) | 11 (3%) |  | 10 (3%) | 11 (3%) |  |
| **Grade** |  |  | <0.001 |  |  | 0.984 |
| Grade III | 226 (23%) | 113 (35%) |  | 113 (35%) | 113 (35%) |  |
| Grade IV | 649 (67%) | 170 (53%) |  | 173 (54%) | 170 (53%) |  |
| Grade II | 74 (8%) | 29 (9%) |  | 27 (8%) | 29 (9%) |  |
| Grade I | 25 (3%) | 9 (3%) |  | 8 (2%) | 9 (3%) |  |
| **Chemotherapy** |  |  | 0.029 |  |  | 0.691 |
| No | 837 (86%) | 291 (91%) |  | 288 (90%) | 291 (91%) |  |
| Yes | 137 (14%) | 30 (9%) |  | 33 (10%) | 30 (9%) |  |
| ^1^n (%) | | | | | | |
| ^2^Pearson's Chi-squared test | | | | | | |

**Table S4B Characteristics of the N+ subgroup before and after PSM stratified by nLNE of 8.**

| **Characteristics** | **Unmatched** | | | **Matched** | | |
| --- | --- | --- | --- | --- | --- | --- |
|  | **≥8**, N = 48^1^ | **＜8**, N = 10^1^ | **p-value**^2^ | **≥8**, N = 10^1^ | **＜8**, N = 10^1^ | **p-value**^3^ |
| **Age** |  |  | 0.487 |  |  | >0.999 |
| ≥65 | 23 (48%) | 6 (60%) |  | 5 (50%) | 6 (60%) |  |
| ＜65 | 25 (52%) | 4 (40%) |  | 5 (50%) | 4 (40%) |  |
| **Sex** |  |  | 0.439 |  |  | >0.999 |
| Male | 36 (75%) | 6 (60%) |  | 7 (70%) | 6 (60%) |  |
| Female | 12 (25%) | 4 (40%) |  | 3 (30%) | 4 (40%) |  |
| **Race** |  |  | 0.274 |  |  | >0.999 |
| White | 44 (92%) | 8 (80%) |  | 9 (90%) | 8 (80%) |  |
| Others | 2 (4%) | 1 (10%) |  | 1 (10%) | 1 (10%) |  |
| Black | 2 (4%) | 1 (10%) |  | 0 (0%) | 1 (10%) |  |
| **Marital.status** |  |  | >0.999 |  |  | >0.999 |
| Married | 28 (58%) | 6 (60%) |  | 7 (70%) | 6 (60%) |  |
| Others | 20 (42%) | 4 (40%) |  | 3 (30%) | 4 (40%) |  |
| **Histology** |  |  | >0.999 |  |  | >0.999 |
| TCC | 46 (96%) | 10 (100%) |  | 10 (100%) | 10 (100%) |  |
| VH | 2 (4%) | 0 (0%) |  | 0 (0%) | 0 (0%) |  |
| **Grade** |  |  | 0.074 |  |  | >0.999 |
| Grade III | 13 (27%) | 2 (20%) |  | 3 (30%) | 2 (20%) |  |
| Grade IV | 33 (69%) | 5 (50%) |  | 5 (50%) | 5 (50%) |  |
| Grade II | 1 (2%) | 2 (20%) |  | 1 (10%) | 2 (20%) |  |
| Grade I | 1 (2%) | 1 (10%) |  | 1 (10%) | 1 (10%) |  |
| **Chemotherapy** |  |  | >0.999 |  |  | >0.999 |
| No | 22 (46%) | 4 (40%) |  | 3 (30%) | 4 (40%) |  |
| Yes | 26 (54%) | 6 (60%) |  | 7 (70%) | 6 (60%) |  |
| ^1^n (%) | | | | | | |
| ^2^Pearson's Chi-squared test; Fisher's exact test | | | | | | |
| ^3^Fisher's exact test | | | | | | |

**Table S4C Characteristics of the octogenarian patients before and after PSM stratified by nLNE of 8.**

| **Characteristics** | **Unmatched** | | | **Matched** | | |
| --- | --- | --- | --- | --- | --- | --- |
|  | **＜8**, N = 112^1^ | **≥8**, N = 79^1^ | **p-value**^2^ | **＜8**, N = 79^1^ | **≥8**, N = 79^1^ | **p-value**^2^ |
| **Age.recode.with..1.year.olds** |  |  | 0.137 |  |  | 0.848 |
| 80-84 years | 77 (69%) | 62 (78%) |  | 61 (77%) | 62 (78%) |  |
| 85+ years | 35 (31%) | 17 (22%) |  | 18 (23%) | 17 (22%) |  |
| **Sex** |  |  | 0.317 |  |  | 0.417 |
| Male | 87 (78%) | 66 (84%) |  | 62 (78%) | 66 (84%) |  |
| Female | 25 (22%) | 13 (16%) |  | 17 (22%) | 13 (16%) |  |
| **Race** |  |  | 0.910 |  |  | 0.893 |
| White | 104 (93%) | 72 (91%) |  | 74 (94%) | 72 (91%) |  |
| Black | 3 (3%) | 2 (3%) |  | 2 (3%) | 2 (3%) |  |
| Others | 5 (4%) | 5 (6%) |  | 3 (4%) | 5 (6%) |  |
| **Marital.status** |  |  | 0.465 |  |  | 0.743 |
| Others | 47 (42%) | 29 (37%) |  | 31 (39%) | 29 (37%) |  |
| Married | 65 (58%) | 50 (63%) |  | 48 (61%) | 50 (63%) |  |
| **Histology** |  |  | 0.279 |  |  | 0.442 |
| TCC | 109 (97%) | 74 (94%) |  | 77 (97%) | 74 (94%) |  |
| VH | 3 (3%) | 5 (6%) |  | 2 (3%) | 5 (6%) |  |
| **Grade** |  |  | 0.110 |  |  | 0.501 |
| Grade II | 15 (13%) | 3 (4%) |  | 0 (0%) | 3 (4%) |  |
| Grade IV | 58 (52%) | 49 (62%) |  | 51 (65%) | 49 (62%) |  |
| Grade III | 37 (33%) | 26 (33%) |  | 26 (33%) | 26 (33%) |  |
| Grade I | 2 (2%) | 1 (1%) |  | 2 (3%) | 1 (1%) |  |
| **Chemotherapy** |  |  | 0.557 |  |  | 0.755 |
| No | 106 (95%) | 73 (92%) |  | 74 (94%) | 73 (92%) |  |
| Yes | 6 (5%) | 6 (8%) |  | 5 (6%) | 6 (8%) |  |
| **T.stage** |  |  | 0.098 |  |  | 0.739 |
| T1 | 89 (79%) | 71 (90%) |  | 68 (86%) | 71 (90%) |  |
| Ta | 20 (18%) | 6 (8%) |  | 8 (10%) | 6 (8%) |  |
| Tis | 3 (3%) | 2 (3%) |  | 3 (4%) | 2 (3%) |  |
| ^1^n (%) | | | | | | |
| ^2^Pearson's Chi-squared test; Fisher's exact test | | | | | | |


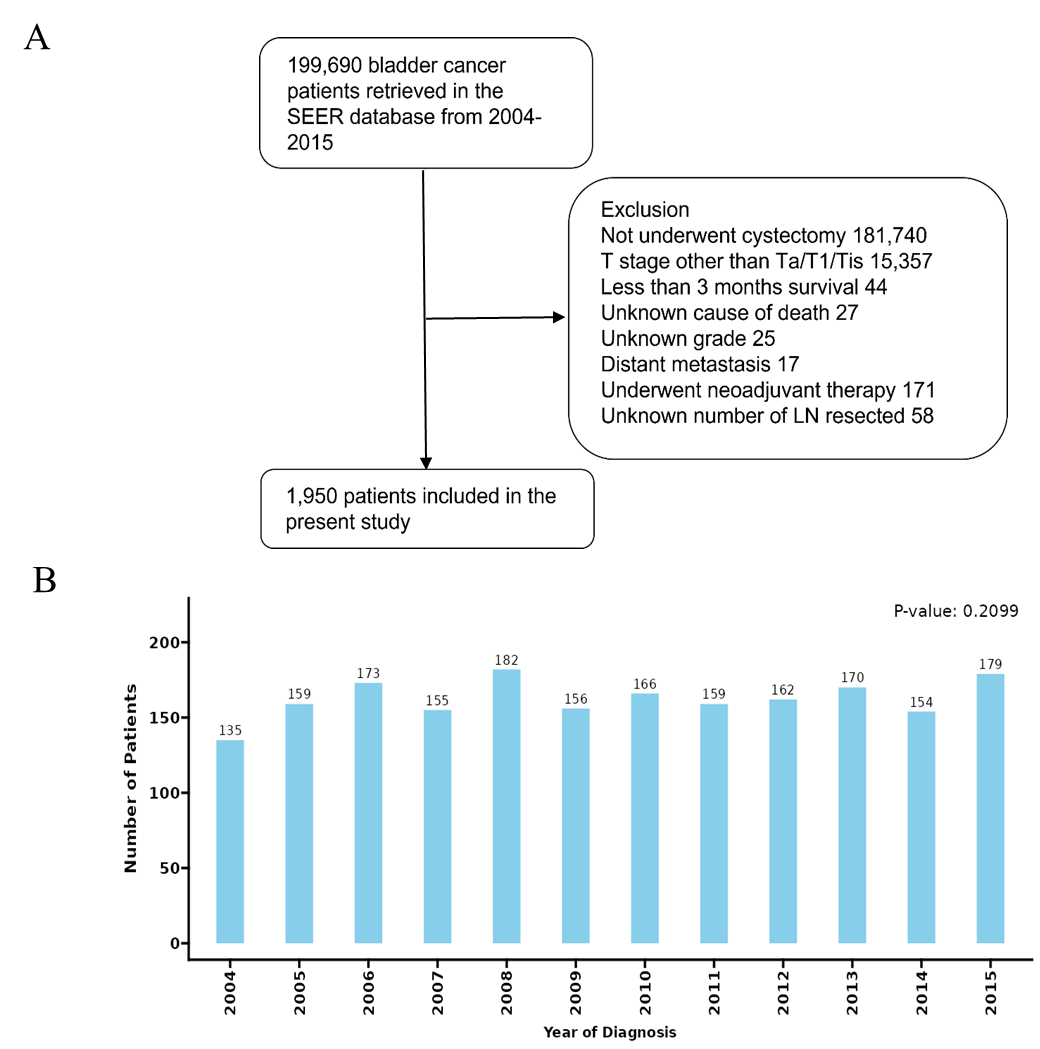
 **Figure S1.** (A) Flowchart of patient selection; (B) Trends in the number of patients undergoing Cystectomy for NMIBC in the SEER registry.

A B


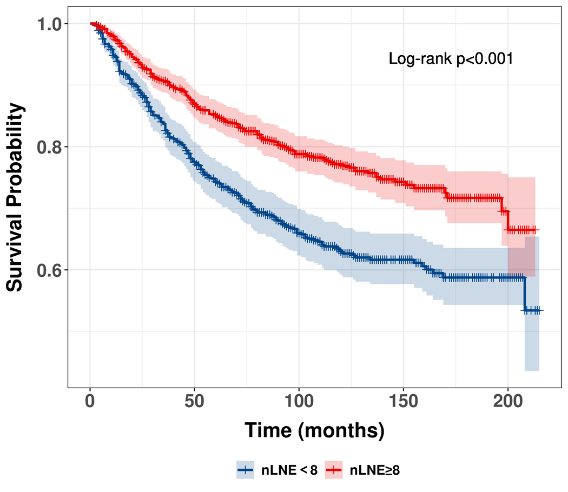

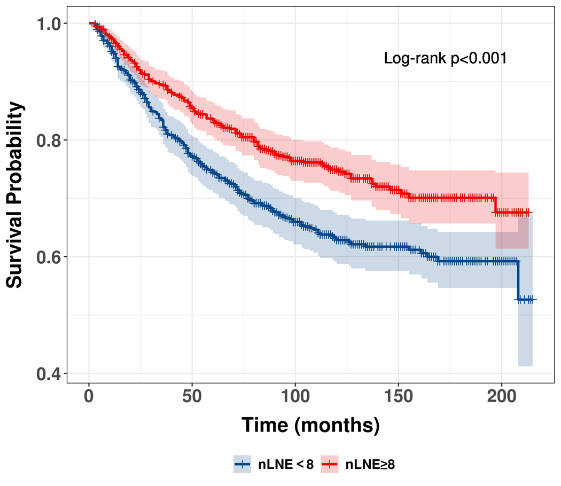


C D


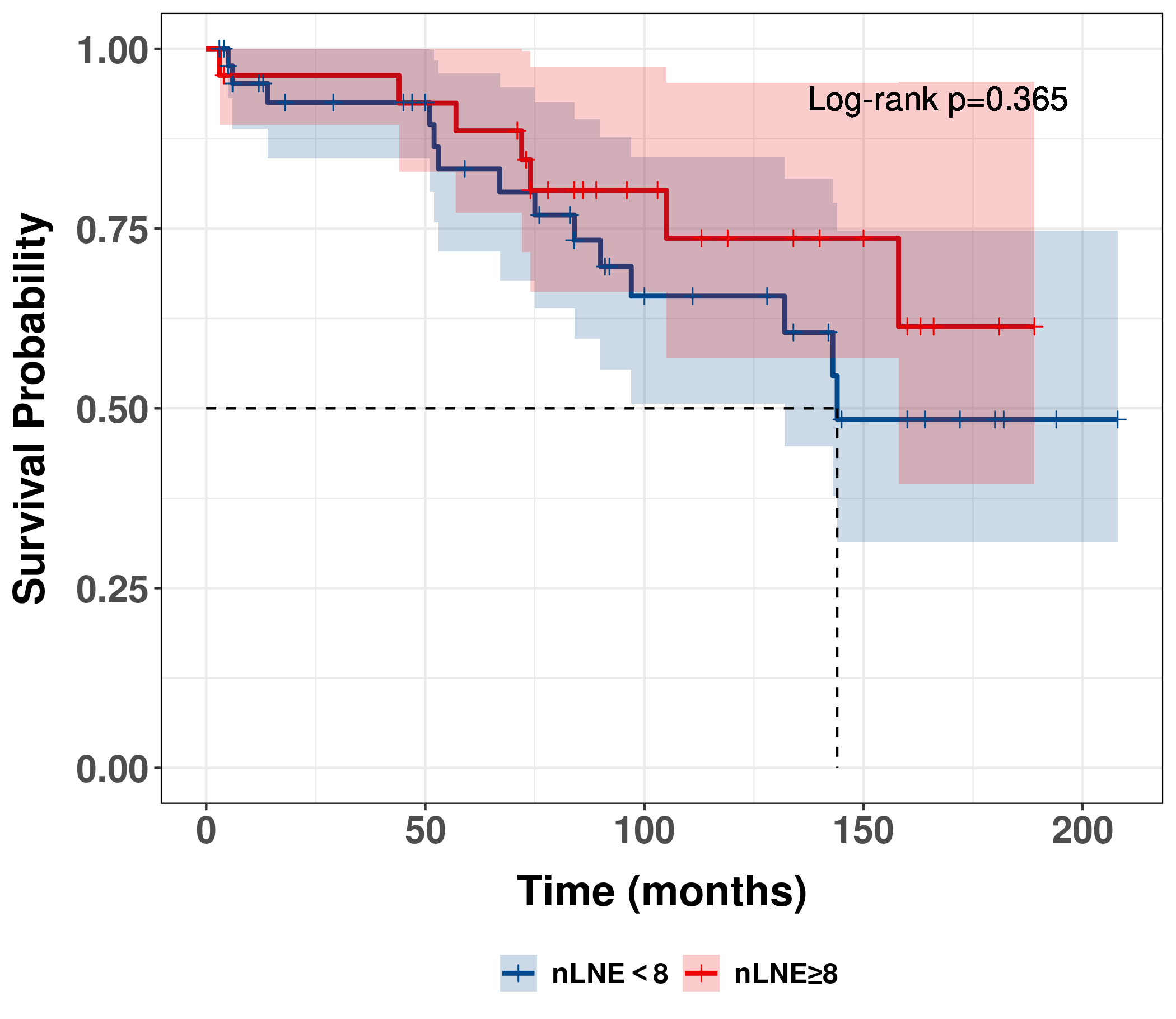

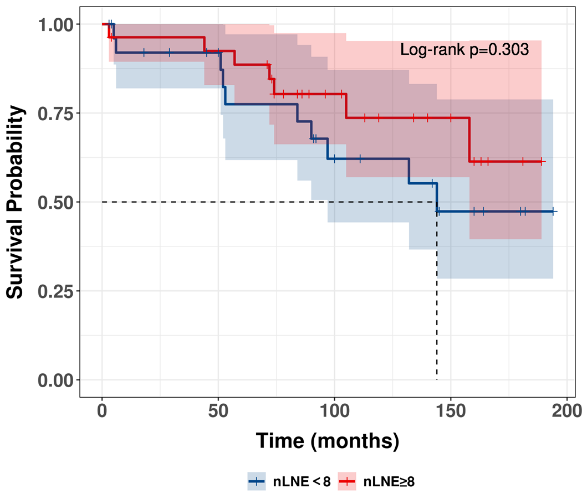


E


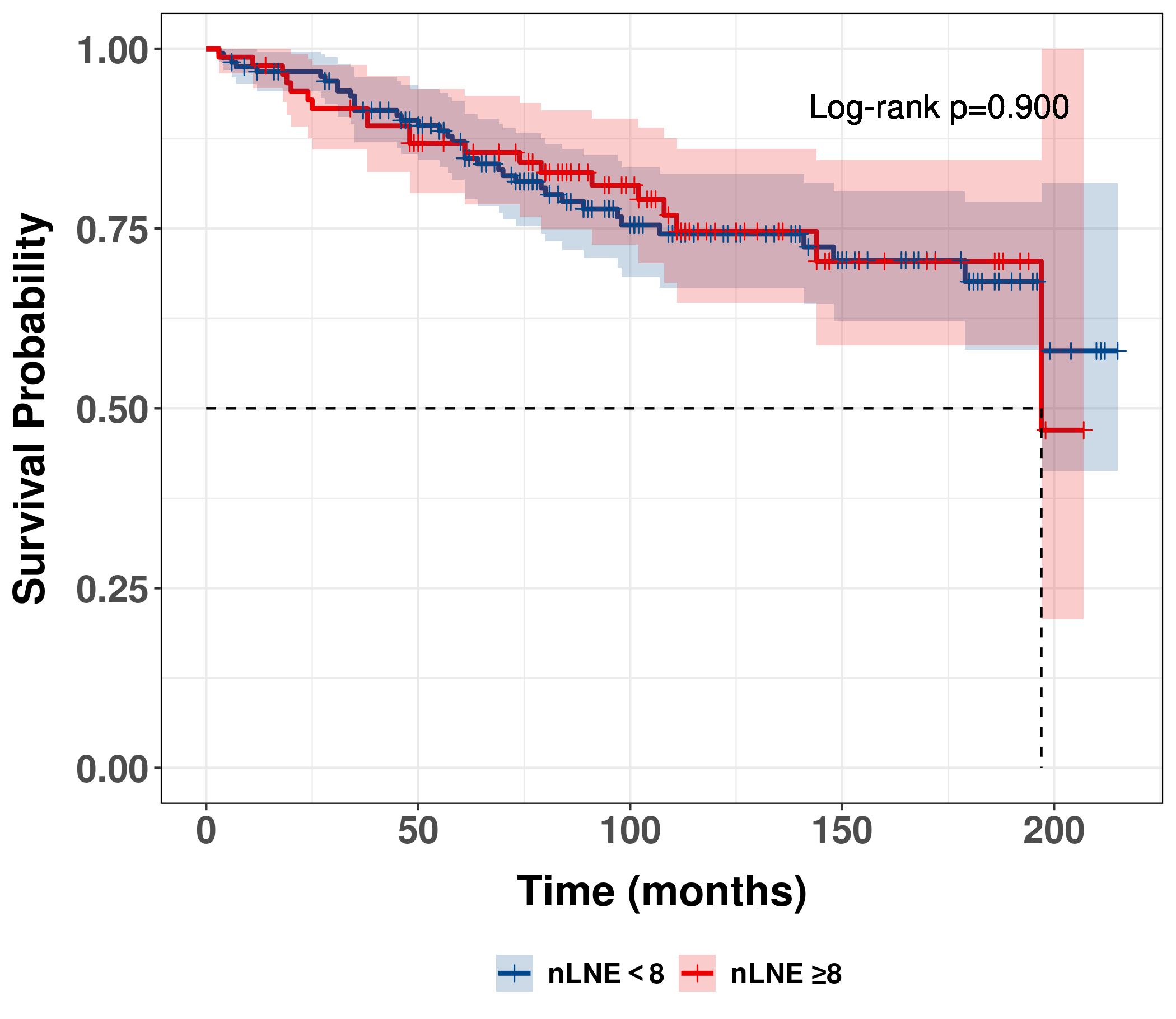

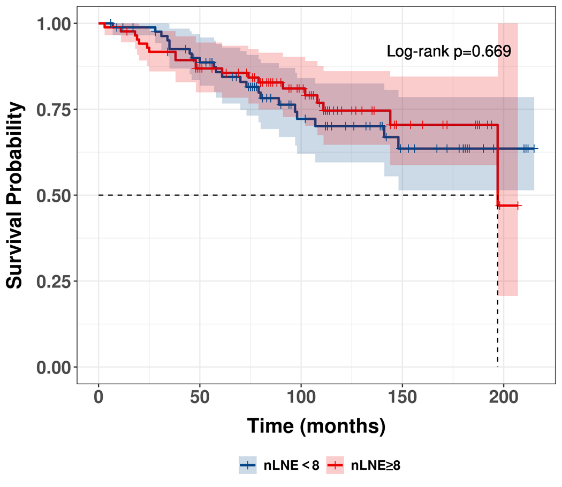


G H


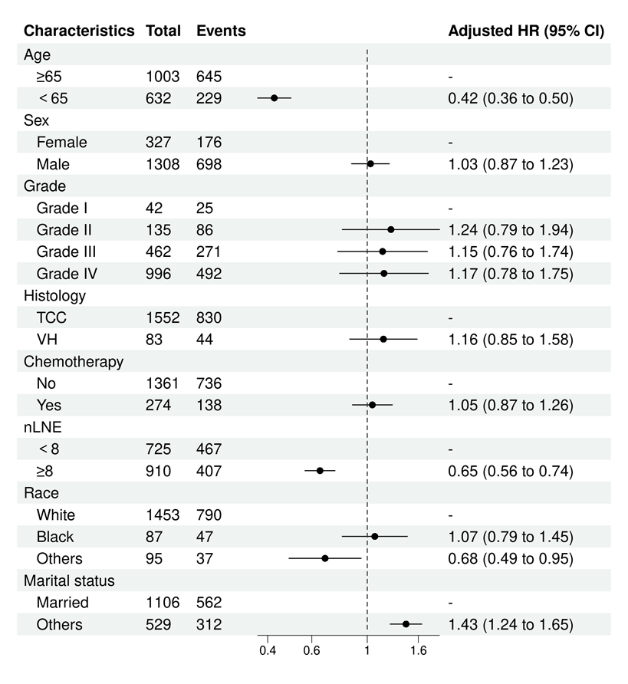
**
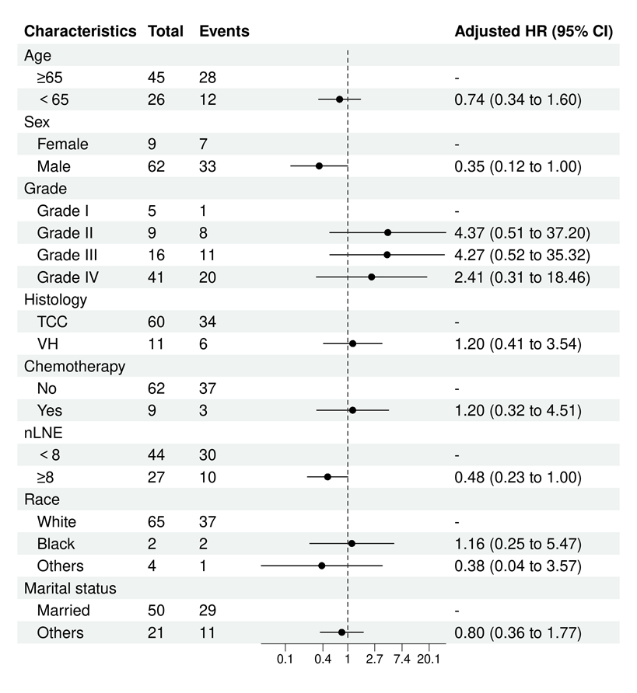
**

I


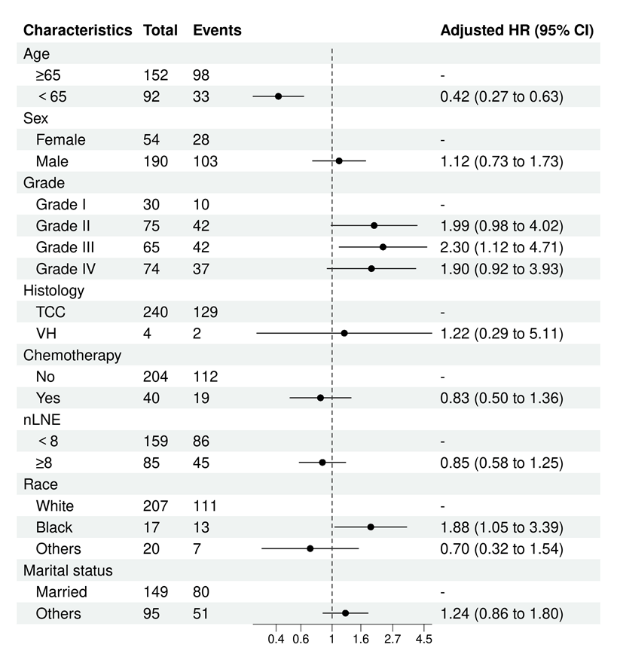


**Figure S2.** Prognostic differences between adequate and limited LND for CSS in NMIBC patients. (A-B) K–M curves of T1 patients before (A) and after PSM (B); (C–D) K–M curves of Tis patients before (C) and after PSM (D); (E–F) K–M curves of Ta patients before (E) and after PSM (F). Multivariable cox regression of T1(Figure G), Tis(Figure H) and Ta patients(Figure I).

A B


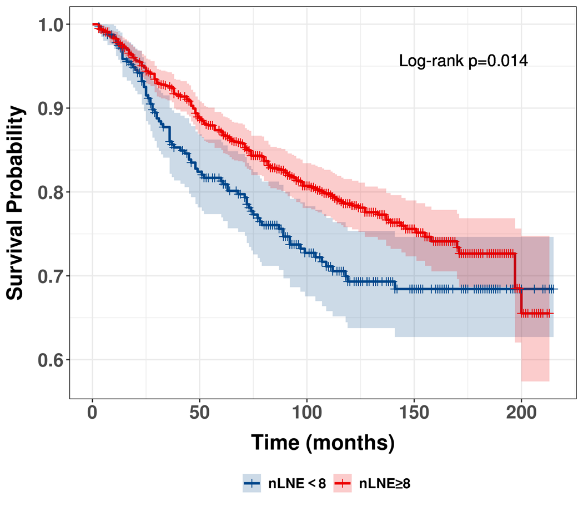

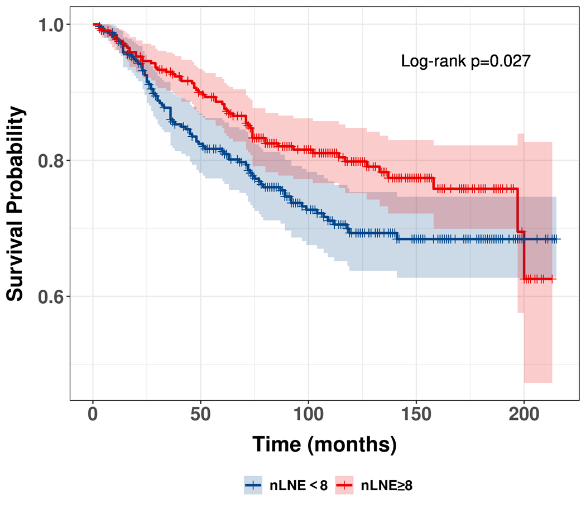


C


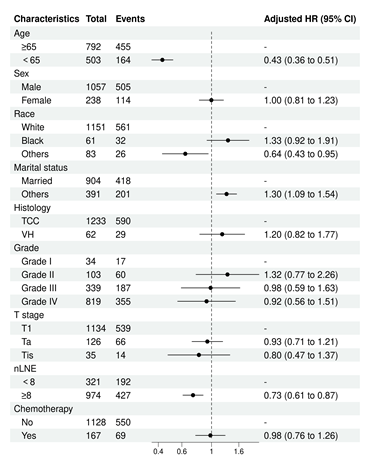


D E


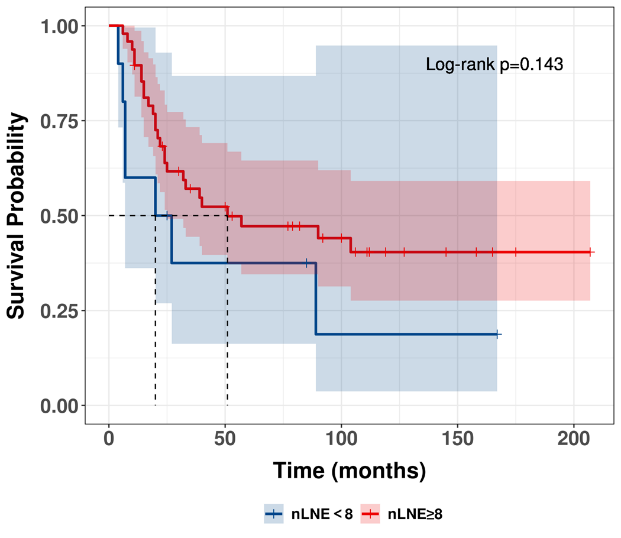

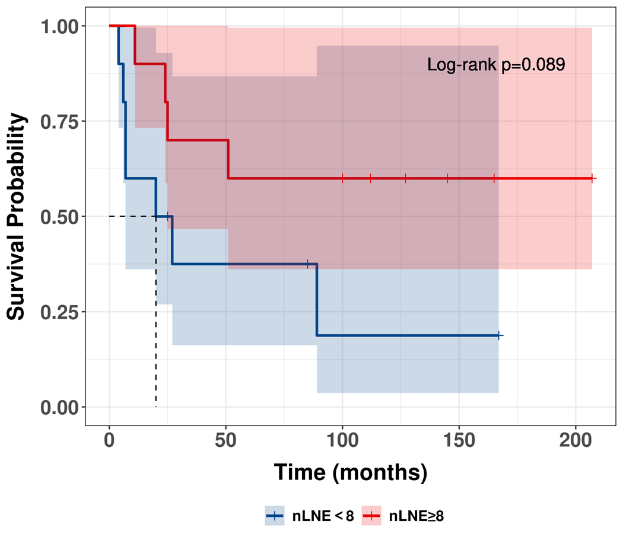


F


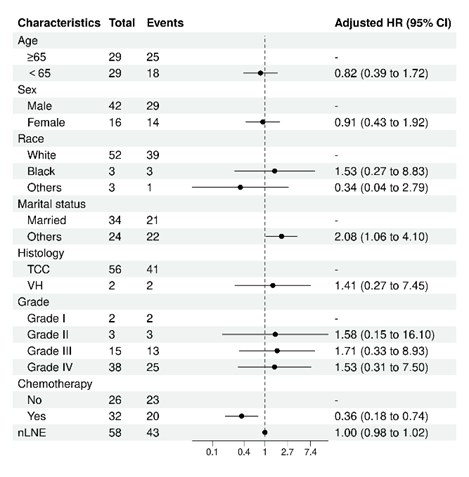


**Figure S3.** Prognostic differences between adequate and limited LND for CSS in NMIBC patients. (A-B) K–M curves of N- patients before (A) and after PSM (B); (C) Multivariable cox regression of N- patients. (D–E) K–M curves of N+ patients before (D) and after PSM (E); (F). Multivariable cox regression of N+ patients.

A B

**
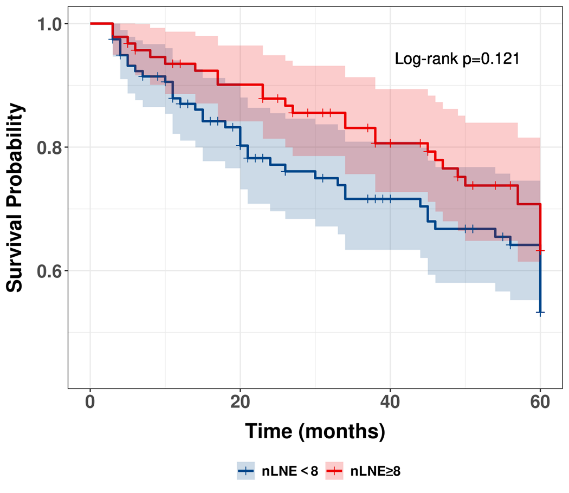

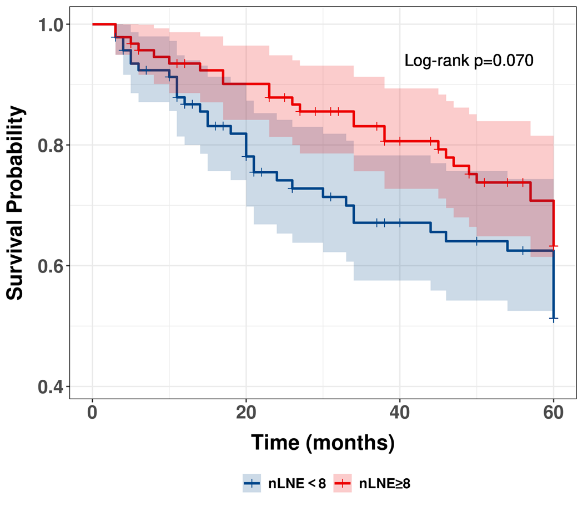
**

**Figure S4.** Sensitivity analysis K–M curves of CSS in octogenarian patients before (A) and after PSM (B)
